# Ferroptosis-related lncRNAs guiding osteosarcoma prognosis and immune microenvironment

**DOI:** 10.1186/s13018-023-04286-3

**Published:** 2023-10-19

**Authors:** Mingyi Yang, Yani Su, Ke Xu, Haishi Zheng, Qiling Yuan, Yongsong Cai, Yirixiati Aihaiti, Peng Xu

**Affiliations:** Department of Joint Surgery, HongHui Hospital, Xi’an Jiaotong University, Xi’an, 710054 Shaanxi China

**Keywords:** Osteosarcoma, Ferroptosis, Gene, LncRNA, Immunity, Prognosis

## Abstract

**Objective:**

To investigate the ferroptosis-related long non-coding RNAs (FRLncs) implicated in influencing the prognostic and immune microenvironment in osteosarcoma (OS), and to establish a foundational framework for informing clinical decision making pertaining to OS management.

**Methods:**

Transcriptome data and clinical data pertaining to 86 cases of OS, the GSE19276, GSE16088 and GSE33382 datasets, and a list of ferroptosis-related genes (FRGs) were used to establish a risk prognostic model through comprehensive analysis. The identification of OS-related differentially expressed FRGs was achieved through an integrated analysis encompassing the aforementioned 86 OS transcriptome data and the GSE19276, GSE16088 and GSE33382 datasets. Concurrently, OS-related FRLncs were ascertained via co-expression analysis. To establish a risk prognostic model for OS, Univariate Cox regression analysis and Lasso Cox regression analysis were employed. Subsequently, a comprehensive evaluation was conducted, comprising risk curve analysis, survival analysis, receiver operating characteristic curve analysis and independent prognosis analysis. Model validation with distinct clinical subgroups was performed to assess the applicability of the risk prognostic model to diverse patient categories. Moreover, single sample gene set enrichment analysis (ssGSEA) was conducted to investigate variations in immune cell populations and immune functions within the context of the risk prognostic model. Furthermore, an analysis of immune checkpoint differentials yielded insights into immune checkpoint-related genes linked to OS prognosis. Finally, the risk prognosis model was verified by dividing the samples into train group and test group.

**Results:**

We identified a set of seven FRLncs that exhibit potential as prognostic markers and influence factors of the immune microenvironment in the context of OS. This ensemble encompasses three high-risk FRLncs, denoted as APTR, AC105914.2 and AL139246.5, alongside four low-risk FRLncs, designated as DSCR8, LOH12CR2, AC027307.2 and AC025048.2. Furthermore, our analysis revealed notable down-regulation in the high-risk group across four distinct immune cell types, namely neutrophils, natural killer cells, plasmacytoid dendritic cells and tumor-infiltrating lymphocytes. This down-regulation was also reflected in four key immune functions, antigen-presenting cell (APC)-co-stimulation, checkpoint, cytolytic activity and T cell co-inhibition. Additionally, we identified seven immune checkpoint-associated genes with significant implications for OS prognosis, including CD200R1, HAVCR2, LGALS9, CD27, LAIR1, LAG3 and TNFSF4.

**Conclusion:**

The findings of this study have identified FRLncs capable of influencing OS prognosis and immune microenvironment, as well as immune checkpoint-related genes that are linked to OS prognosis. These discoveries establish a substantive foundation for further investigations into OS survival and offer valuable insights for informing clinical decision making in this context.

**Supplementary Information:**

The online version contains supplementary material available at 10.1186/s13018-023-04286-3.

## Introduction

Osteosarcoma (OS) stands as the prevailing malignant neoplasm affecting bone tissue, with a predilection for the adolescent demographic [1]. This condition is typified by frequent vascular infiltration, adjacent soft tissue infiltration, a notable proclivity for local recurrence and premature distant metastasis [2]. Approximately one-fifth of OS patients experience the emergence of metastatic lesions, while the remainder often develop subclinical micrometastases. Standard therapeutic modalities encompass the deployment of chemotherapy and surgical resection [3]. Notwithstanding the comprehensive implementation of a multidisciplinary regimen, which encompasses chemotherapeutic intervention and extensive surgical excision, discernible enhancements in clinical outcomes have been documented in patients with OS. However, in instances of advanced disease with remote metastasis and local recurrence, even with the rigorous administration of chemotherapy, clinical outcomes and 5-year overall survival rates remain suboptimal [4]. A burgeoning corpus of scientific inquiry posits that a multifaceted interplay of cellular and molecular events may underlie the pathogenesis of OS [1]. Long non-coding RNA (lncRNA) constitutes a pivotal regulator in a myriad of biological processes, including cell proliferation, apoptosis, invasion and migration. Anomalous expression of lncRNA assumes a pivotal role in the orchestration of tumoral metastasis [5, 6]. The nexus between lncRNA and OS is undeniably close, as investigations have unearthed compelling evidence. For instance, lncRNA LOC100129620 has been implicated in the promotion of OS progression through the modulation of cyclin dependent kinase 6 (CDK6) expression, tumor angiogenesis and macrophage polarization [5]. Conversely, LncRNA FTX exerts an inhibitory effect on OS proliferation and migration via its regulatory influence on the miR-320a/TXNRD1 axis [6]. The exploration of the interrelationship between lncRNA and OS bears paramount significance in the context of disease prognosis and therapeutic intervention. Consequently, the quest for novel prognostic markers in the realm of OS and the pursuit of strategies to enhance clinical efficacy remain imperatives in the management of this disease.

Ferroptosis constitutes a tightly regulated mode of cellular demise, triggered by perturbations in the intracellular milieu, primarily governed by the activity of glutathione peroxidase 4 (GPX4) [7]. The pivotal drivers of ferroptosis initiation encompass the accrual of ferrous iron (Fe^2+^) and the subsequent peroxidation of lipids [7]. Notably, ferroptosis is amenable to inhibition via iron-chelating agents and lipophilic antioxidants [7]. This multifaceted cellular event intricately interweaves processes inclusive of iron homeostasis, lipid metabolism, oxidative stress, as well as the synthesis of essential molecules such as nicotinamide adenine dinucleotide phosphate (NADPH), glutathione (GSH) and coenzyme Q10 (CoQ10) [8]. Accumulating investigations have underscored the pivotal role of ferroptosis in various malignancies. In the context of breast cancer cells, both a lysosome-disrupting agent, silamesine and a tyrosine kinase inhibitor, lapatinib, have been demonstrated to induce ferroptosis [9]. Furthermore, in the realm of pancreatic ductal adenocarcinoma (PDAC), the agent artesunate (ART) exerts its ferroptosis effects through the generation of reactive oxygen species (ROS) in an iron-dependent manner [10]. These findings collectively emphasize the burgeoning potential of ferroptosis as a therapeutic modality in the domain of oncology, consequently intensifying research efforts toward the design and development of anticancer agents capable of inducing ferroptosis [11]. Moreover, an emerging intersection has been observed between ferroptosis and OS. For instance, tirapazamine has been shown to exert partial inhibition of OS cells through the mediation of solute carrier family 7 member 11 (SLC7A11)-associated ferroptosis [12]. Similarly, EF24 has been identified as an inducer of ferroptosis in OS cells, operating via heme oxygenase 1 (HMOX1)-dependent mechanisms [13]. Furthermore, the identification and characterization of ferroptosis-related genes (FRGs) capable of prognosticating outcomes in OS patients have constituted pivotal advancements in this realm of study [14].

**Fig. 1:**
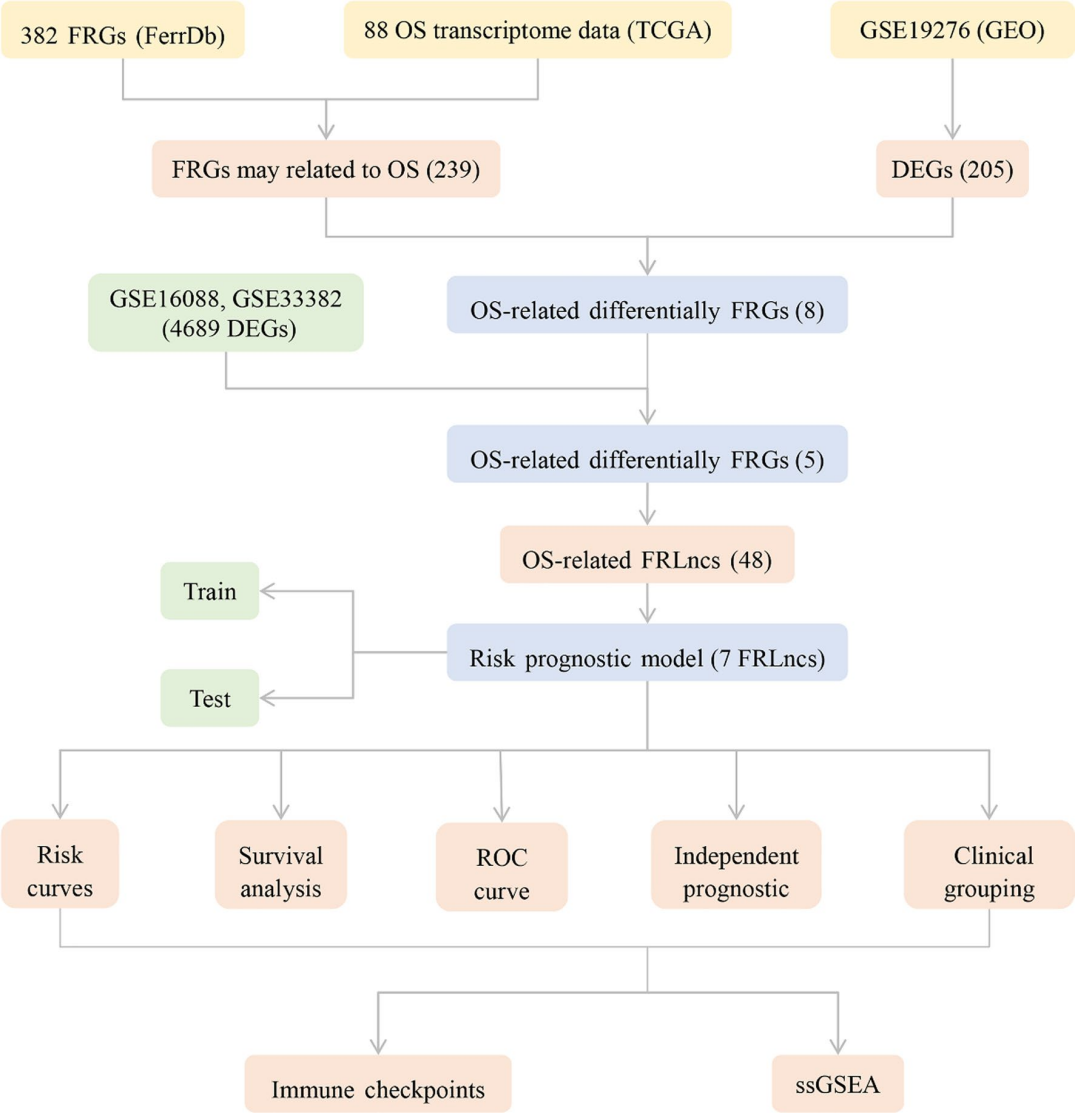
The analysis flowchart of this study

Bioinformatics represents an emergent interdisciplinary field that amalgamates principles from molecular biology and information technology. This convergence bears considerable significance in elucidating the molecular underpinnings of diseases [15]. In recent years, numerous prognostic models for tumors, grounded in the analysis of FRGs, have been successfully devised. Such models hold considerable promise in prognosticating tumor outcomes and facilitating the development of molecularly targeted therapeutic agents [16]. In the present investigation, we applied bioinformatics techniques to construct a prognostic model for OS predicated upon ferroptosis-related lncRNAs (FRLncs). This not only involved an exploration of the prognostic implications of FRLncs in OS patients but also an investigation into their potential associations with the immunological microenvironment in the context of OS. The analysis flowchart of this study is shown in Fig. 1.

## Materials and methods

### Data download and arrangement

The transcriptome data pertaining to 86 cases of OS and accompanying clinical information were obtained from The Cancer Genome Atlas (TCGA) database, accessible at https://portal.gdc.cancer.gov/. The OS transcriptome dataset encompassed both messenger RNA (mRNA) and lncRNA data. The clinical dataset includes futime, fustat, gender, age at diagnosis in days, metastatic status (metastatic/non-metastatic), primary tumor site and specific tumor site. Additionally, the GSE19276, GSE16088 and GSE33382 datasets were procured from the Gene Expression Omnibus (GEO) database, available at https://www.ncbi.nlm.nih.gov/geo/. The GSE19276 dataset encompasses five normal tissue samples and 23 OS tissue samples. The GSE16088 dataset comprised six normal tissue samples and 14 OS tissue samples, while the GSE33382 dataset included three normal tissue samples and 84 OS tissue samples. Furthermore, a comprehensive list of FRGs was sourced from the FerrDb database, accessible at http://www.zhounan.org/ferrdb/.

### OS-related differentially FRGs

The list of FRGs was intersected with the gene set from the OS transcriptome data, yielding a set of FRGs may be related to OS. The original study of GSE19276 dataset obtained 205 differentially expressed genes (DEGs) through performed differential analysis between five normal tissue samples and 23 OS tissue samples. The screening criteria for difference analysis were as follows: *P* < 0.05 and |logFC|≥ 1 [17]. Differential analysis of the GSE16088 and GSE33382 datasets was conducted utilizing the “limma” package in the *R* programming environment. DEGs in OS were identified based on stringent criteria, with a significance threshold of *P* < 0.05 and |logFC|≥ 1 [15]. The FRGs may be related to OS, DEGs from GSE19276 dataset, and DEGs of GSE16088 and GSE33382 datasets were subsequently intersected, resulting in the identification of OS-related differentially expressed FRGs.

### OS-related FRLncs

The co-expression analysis of OS-related differentially expressed FRGs and lncRNAs within the OS transcriptome dataset was conducted utilizing the “limma” package in the *R*. The objective of this analysis was to identify co-expression lncRNAs of OS-related differentially expressed FRGs, herein referred to as OS-related FRLncs. The criteria for screening were established as follows: |Person correlation coefficient|> 0.4, *P* < 0.001, as indicated in a previous study [18]. Cytoscape [19] visualizes the one-to-one correspondence between OS-related differentially expressed FRGs and co-expression lncRNAs.

### Construction of risk prognostic model

Perl integrated expression matrices of OS-related FRLncs with OS survival data. The “survival” package in the *R* was employed to identify statistically significant FRLncs that are associated with OS prognosis, utilizing Univariate Cox regression analysis. To mitigate the potential issue of overfitting, the “glmnet” package in *R* was utilized to conduct Lasso Cox regression analysis. Construct a risk prognosis model for OS prognosis FRLncs. The main body of the risk prognosis model is the optimal number of FRLncs obtained by Lasso Cox regression analysis. A riskscore for each sample was calculated based on these FRLncs. The risk prognosis model divides the sample into two groups: high-risk and low-risk groups. The essence of the risk prognosis model is to compare the survival differences of patients with OS between high-risk and low-risk groups. Calculate the riskscore: Riskscore = ${\sum }_{i=1}^{n}\left(lncrnaex{p}_{i}\times coe{f}_{i}\right)$, n represents the number of OS prognostic FRLncs, i is the ith FRLncs, and coef is the regression coefficient. Each OS prognostic FRLncs's expression level is multiplied by its corresponding regression coefficient, and then accumulated to obtain the sample riskscore. Subsequently, the samples are dichotomized into high-risk and low-risk groups based on the median value of the sample riskscore [20].

### Risk curves and survival analysis

R further generates survival status maps and risk heatmaps to assess differences in survival time and OS prognosis FRLncs among high- and low-risk groups defined by the risk prognostic model [21]. The creation of survival curves, aimed at evaluating potential survival disparities between these high- and low-risk groups within the risk prognostic model, was facilitated using the “survival” and “survminer” packages in *R*.

### Receiver operating characteristic curve (ROC) analysis and independent prognostic analysis

The ROC curves were generated employing the “survival,” “survminer” and “timeROC” packages within the *R*. Utilizing the “survival” package in *R*, we conducted independent prognostic assessments via both univariate and multivariate COX regression analyses. The aim was to investigate the viability of the riskscore within the prognostic model as an autonomous prognostic determinant [22].

### Model validation for clinical grouping

To evaluate the applicability of the risk prognostic model across diverse clinical patient cohorts, we conducted a comprehensive validation procedure involving the alignment of clinical characteristics with the risk prognostic model. The clinical attributes were stratified as follows: gender dichotomized into male and female categories; age categorized into two groups, namely, individuals aged ≤ 5245(14 years old) and > 5245(14 years old); metastasis status categorized as either metastatic or non-metastatic; primary tumor site classified as upper limb or lower limb + pelvis; and specific tumor site categorized as upper limb or lower limb + pelvis. Subsequently, Perl scripting was employed to merge the categorized clinical data with the corresponding riskscores. To assess the model’s suitability for each specific clinical trait, we leveraged the “survival” and “survminer” packages within the *R*, conducting a model validation process for each of the clinical subgroups.

### Single sample gene set enrichment analysis (ssGSEA)

The enrichment scores for immune cells and immune functions in the OS transcriptome data were computed utilizing the *R* packages “GSVA,” “limma” and “GSEABase.” Subsequently, an analysis of differences between high- and low-risk groups in terms of immune cell composition and immune function was performed using the R packages “limma,” “reshape2” and “ggpubr”[20].

### Differential analysis of immune checkpoints

The analysis of correlation among immune checkpoint-related genes and risk prognostic model was conducted using the R packages “limma,” “reshape2,” “ggplot2” and “ggpubr.” The objective was to assess the difference in immune checkpoint-related genes between high- and low-risk groups [22].

### Validation of the risk prognosis model

Importantly, we sought to validate the constructed risk prognosis model. To do so, we evenly partitioned the samples into two distinct groups: a train group and a test group. This partitioning allowed us to assess the model's accuracy effectively. Subsequently, we calculated the riskscore for each sample within the train group, employing the previously described formula. The samples in the train group were then classified into high-risk and low-risk groups based on the median value of riskscore. For the categorization of samples within the test group, we employed a similar approach, dividing them into high-risk and low-risk groups based on the median value of riskscores calculated within the train group. To perform survival analysis, we utilized the “survival” and “survminer” packages of *R*. Subsequently, we employed the *R* to generate a risk heatmap.

## Results

### OS-related differentially FRGs

A total of 382 FRGs were extracted from FerrDb (Additional file 1: Table 1). The transcriptome data of OS from the TCGA database contained 19,262 genes. The intersection of 382 FRGs and 19,262 genes that yielded 239 FRGs may be related to OS. The original study of the GSE19276 dataset provided 205 DEGs (Additional file 1: Table 2).

**Fig. 2:**
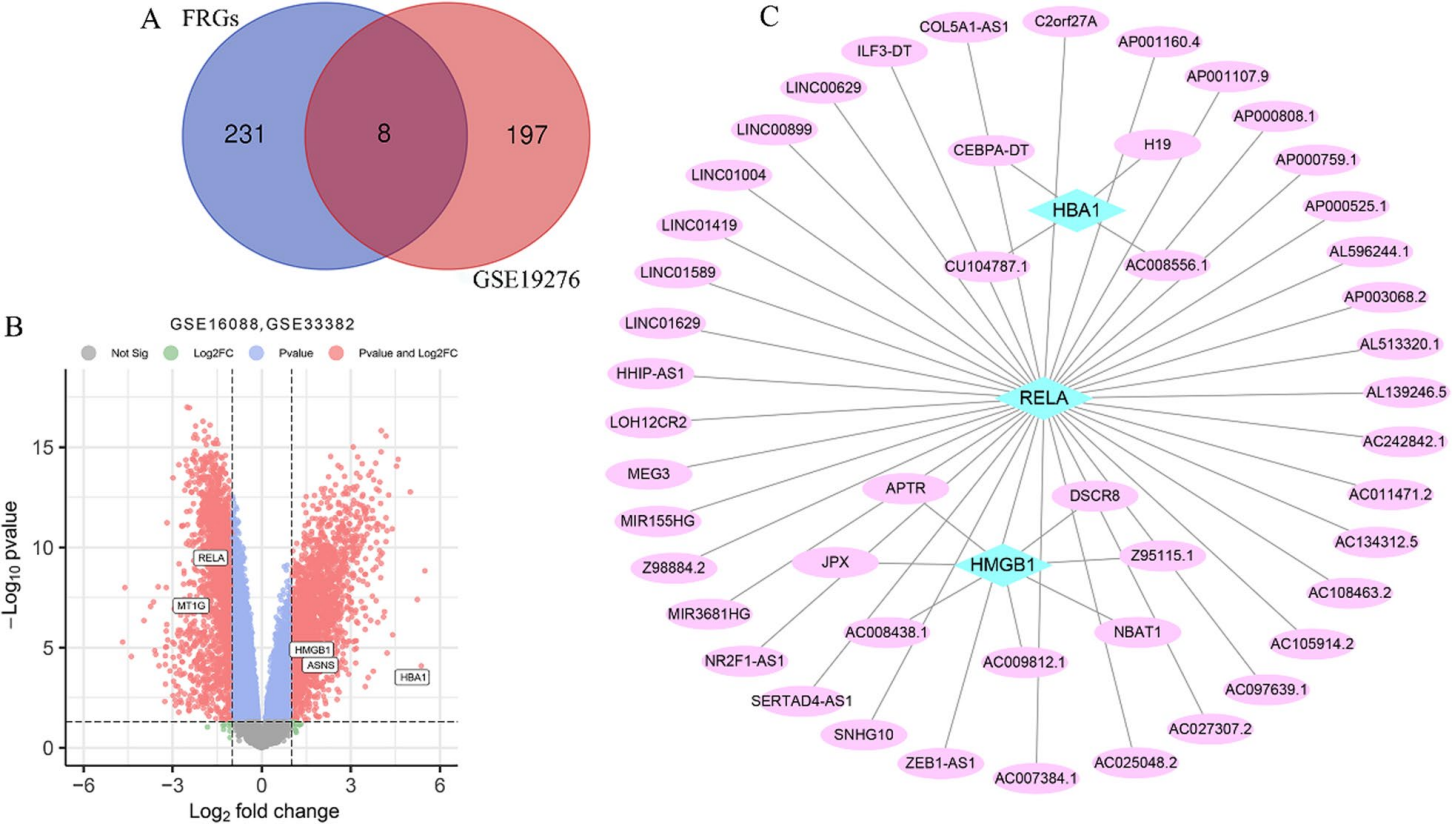
OS-related differentially expressed FRGs and OS-related FRLncs. **a** The intersection of DEGs from GSE19276 dataset and FRGs may be related to OS that obtained OS-related differentially expressed FRGs. **b** Volcanomap of GSE16088 and GSE3338 datasets difference analysis, five OS-related differentially expressed FRGs overlap with DEGs of GSE16088 and GSE3338 datasets. **c** By co-expression analysis, OS-related differentially expressed FRGs yielded a total of 48 OS-related FRLncs. The screening criteria are: |Person correlation coefficient|> 0.4, *P* < 0.001

The overlap analysis between the 239 FRGs and the 205 DEGs from GSE19276 dataset may be related to OS that revealed eight unique genes, referred to as OS-related differentially expressed FRGs **(**Fig. 2a). Subsequently, utilizing a differential analysis of GSE16088 and GSE3338 datasets, a total of 4689 DEGs were identified (Additional file 1: Table 3). These DEGs were subjected to data visualization using the *R*, resulting in the generation of a volcanomap (Fig. 2b). The overlap analysis of eight OS-related differentially expressed FRGs and 4689 DEGs of GSE16088 and GSE3338 datasets further obtained five OS-related differentially expressed FRGs (Fig. 2b).

### OS-related FRLncs

Subsequently, we analyzed the co-expression of five OS-related differentially expressed FRGs and lncRNAs contained in the OS transcriptome data. Through co-expression analysis, a subset consisting of three out of the five OS-related differentially expressed FRGs produced a combined total of 48 OS-related FRLncs (Fig. 2c).

### Construction of risk prognostic model

**Fig. 3:**
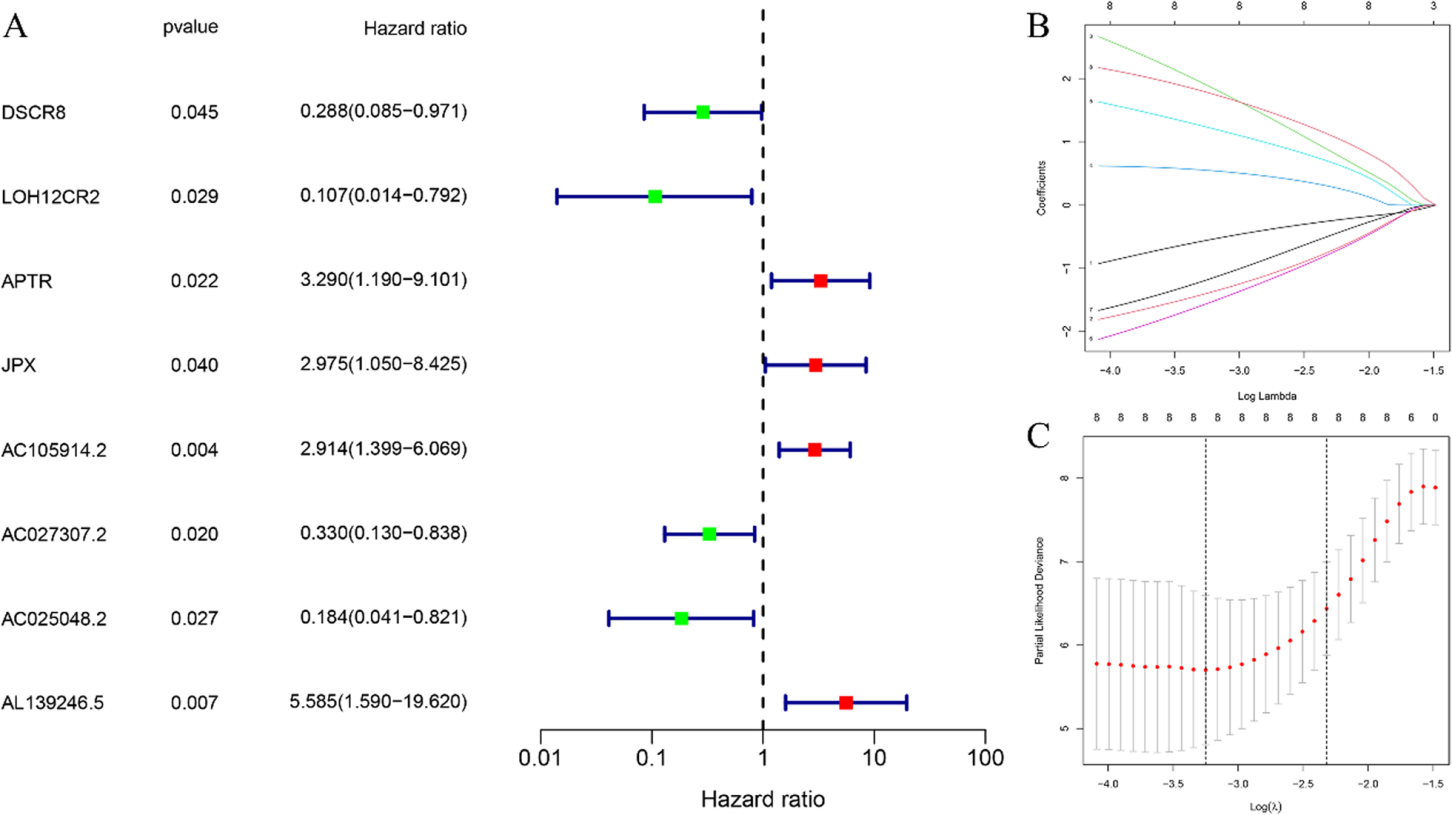
Construction of risk prognostic model. **a** Univariate Cox regression analysis obtained eight candidate prognostic FRLncs for OS, including four high-risk FRLncs and four low-risk FRLncs. **b** LASSO Cox regression analysis for eight candidate prognostic FRLncs. **c** Selection of the optimal penalty parameter for LASSO regression

We employed the expression levels of 48 FRLncs associated with OS as continuous variables and conducted Univariate Cox regression analysis to estimate the hazard ratio (HR). Selection criteria for significance were set at *P* < 0.05. Consequently, we identified a total of eight FRLncs as prognostically relevant candidates for OS, comprising four categorized as high-risk FRLncs and four as low-risk FRLncs (Fig. 3a). Notably, higher expression levels of high-risk FRLncs were associated with elevated patient risk, whereas increased expression of low-risk FRLncs was associated with reduced patient risk. Subsequently, we performed Lasso Cox regression analysis on these eight OS prognostic FRLncs, determining an optimal subset of seven FRLncs based on the optimal penalty parameter (*λ*) value (Fig. 3b, c). Subsequently, we calculated a riskscore for each sample according to the prognostic model formula and stratified patients into high-risk (*N* = 43) and low-risk (*N* = 43) groups based on the median value.

### Risk curves and survival analysis

**Fig. 4:**
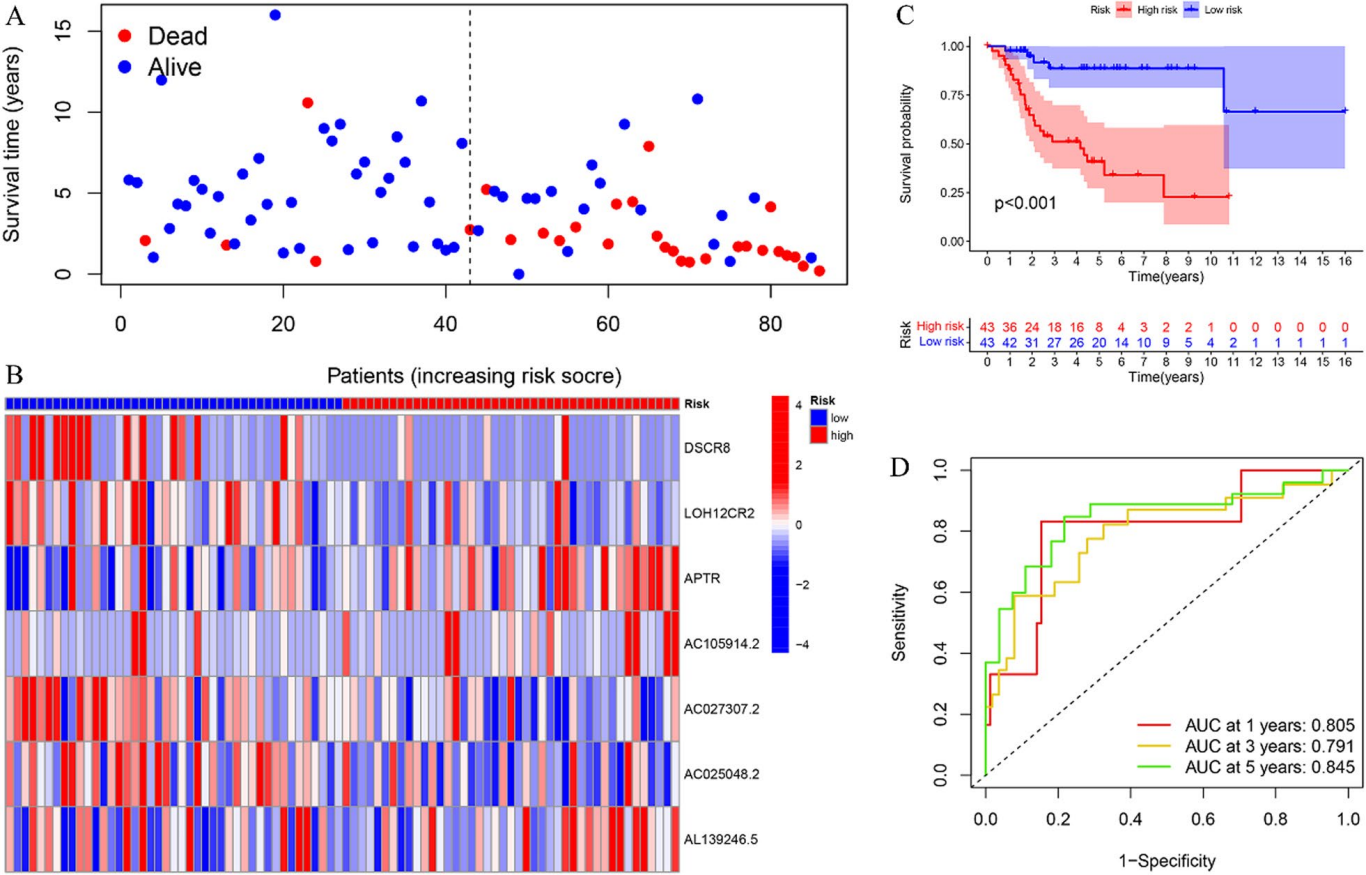
Comprehensive analysis of risk prognosis model. **a** Survival status plot, from the low-risk group to the high-risk group, the survival time of patients with OS decreased. **b** Risk heatmap. APTR, AC105914.2 and AL139246.5 are high-risk FRLncs. DSCR8, LOH12CR2, AC027307.2 and AC025048.2 are low-risk FRLncs **c** Survival analysis, there was a significant survival difference of patients with OS between the high- and low-risk groups. **d** ROC curves, the risk prognosis model could well predict the survival rate of patients with OS at one, three and 5 years

The survival status diagram demonstrates a progressive decrease in the survival rates of patients transitioning from the low-risk group to the high-risk group, concomitant with a corresponding increase in mortality rates (Fig. 4a). Notably, the risk heatmap illuminates the differential expression patterns of specific FRLncs across these risk groups. Specifically, the expressions of APTR, AC105914.2 and AL139246.5exhibit a gradual augmentation from the low-risk to the high-risk group, classifying them as high-risk FRLncs. Conversely, the expression levels of DSCR8, LOH12CR2, AC027307.2 and AC025048.2demonstrate a consistent decline, signifying their classification as low-risk FRLncs (Fig. 4b). Furthermore, survival curve analysis underscores a statistically significant difference in survival outcomes between the high-risk and low-risk groups (*P* < 0.001) (Fig. 4c).

### ROC curve analysis and independent prognostic analysis

**Fig. 5:**
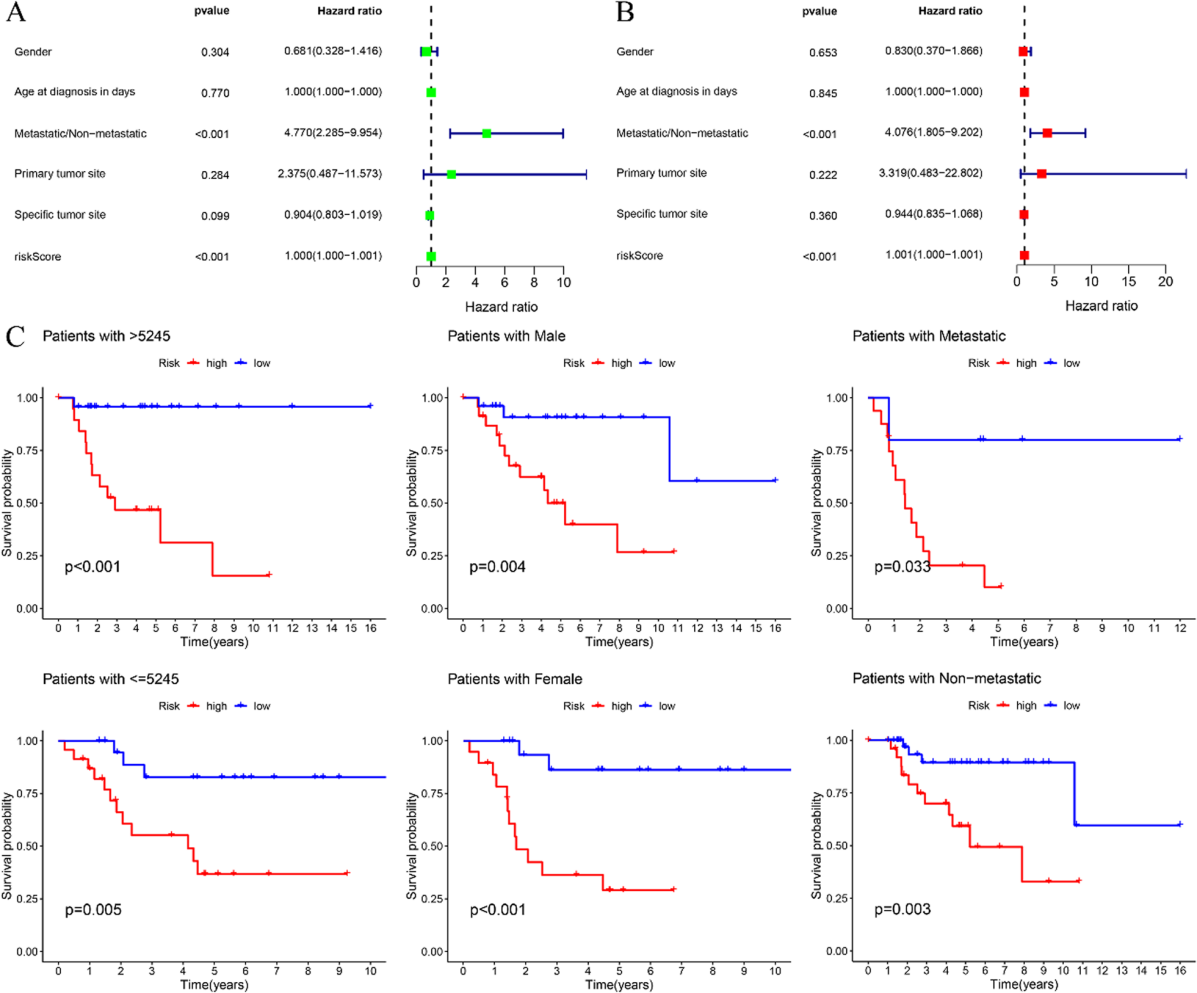
Independent prognostic analysis and model validation for clinical grouping. **a** Univariate independent prognostic analysis, riskscore and metastasis were independent prognostic factors. **b** Multivariate independent prognosis analysis, riskscore and metastasis were independent prognostic factors. **c** Model validation for clinical grouping, the risk prognostic model across various stratifications, encompassing age, gender and tumor metastasis

The ROC curves revealed that the area under the curve (AUC) exhibited higher values at the one-year (AUC = 0.805), three-year (AUC = 0.791) and five-year (AUC = 0.845) time points (Fig. 4d). The ROC curves results showed that the risk prognosis model could well predict the survival rate of patients with OS at one, three and five years. This observation underscores the prognostic utility of the risk model in accurately predicting overall survival. Both univariate independent prognostic analysis (riskscore: *P* < 0.001, tumor metastasis: *P* < 0.001) and multivariate independent prognosis analysis (riskscore: *P* < 0.001, tumor metastasis: *P* < 0.001) consistently demonstrated that the riskscore and tumor metastasis can serve as independent prognostic factors, both associated with a heightened risk of adverse outcomes (Fig. 5a, b).

### Model validation for clinical grouping

The clinical traits were grouped to observe whether the risk prognostic model was suitable for patients in different clinical groups. Model validation for clinical categorizations demonstrated the appropriateness of the risk prognostic model across various stratifications, encompassing age, gender and tumor metastasis (Fig. 5c).

### Single sample gene set enrichment analysis (ssGSEA)

**Fig. 6:**
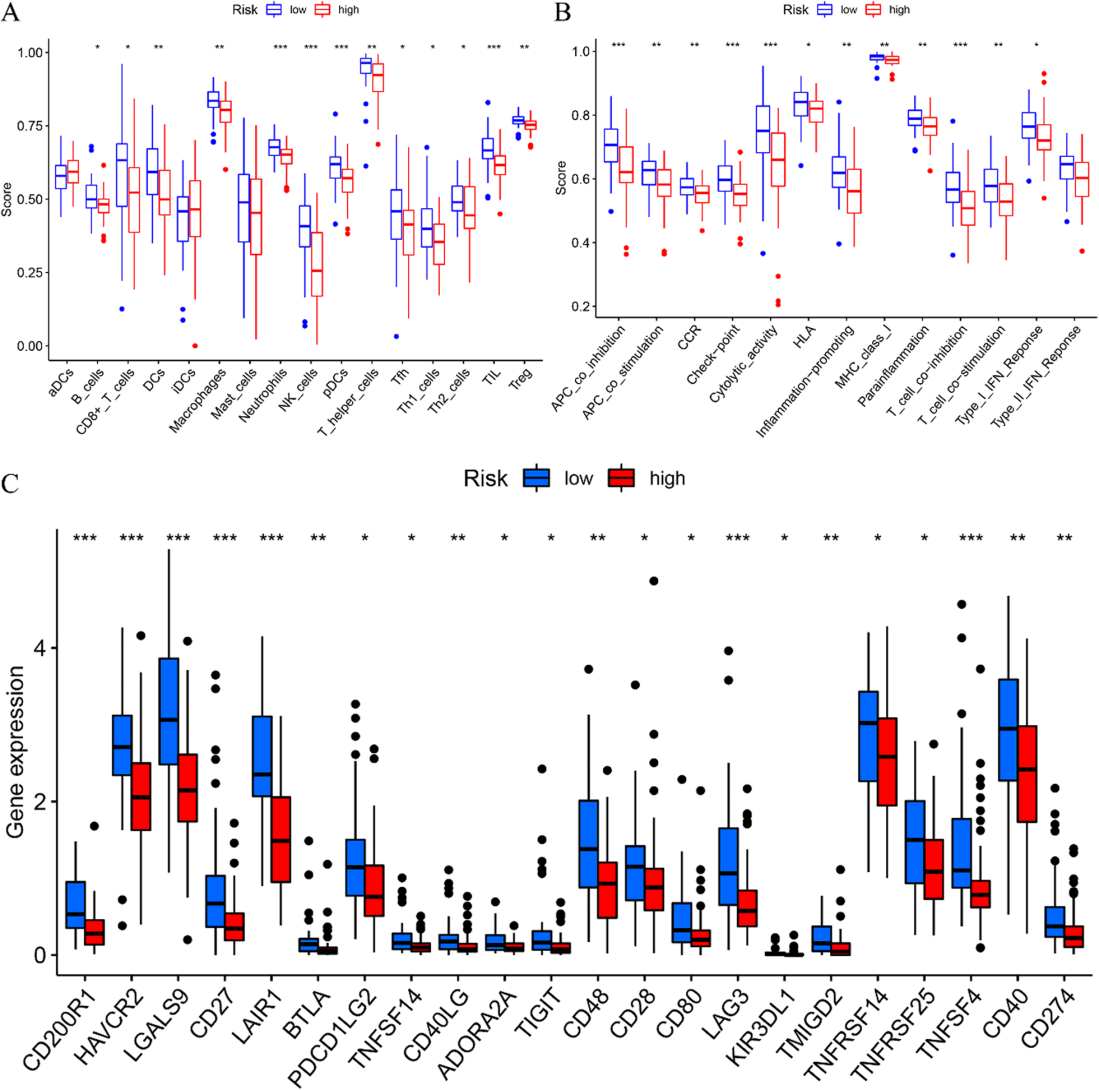
Single sample gene set enrichment analysis and differential analysis of immune checkpoint. **a** Differential analysis of immune cells in high- and low-risk groups in risk prognostic model. **b** Differential analysis of immune function in high- and low-risk groups in risk prognostic model. **c** Differential analysis of immune checkpoint-related genes in high- and low-risk groups in risk prognostic model. * means *P* < 0.05, ** means *P* < 0.01, *** means *P* < 0.001

The differential analysis of immune cell populations within risk prognostic model revealed a notable down-regulation of various immune cell types in the high-risk group (Fig. 6a). These included neutrophils, natural killer (NK) cells, plasmacytoid dendritic cells (pDCs) and tumor-infiltrating lymphocytes (TIL). Furthermore, an examination of immune function within risk prognostic model indicated a significant down-regulation of some immunological processes, specifically, antigen-presenting cell (APC)-co-stimulation, checkpoint, cytolytic activity and T cell co-inhibition, in the high-risk group (Fig. 6b).

### Differential analysis of immune checkpoints

Immune checkpoint differential analysis revealed disparities in the expression levels of 22 immune checkpoint-associated genes between high-risk and low-risk groups. Notably, among these genes, hypocretin receptor 2 (CD200R1), hepatitis A virus cellular receptor 2 (HAVCR2), galectin-9 (LGALS9), cluster of differentiation 27 (CD27), leukocyte associated immunoglobulin like receptor 1 (LAIR1), lymphocyte activating 3 (LAG3) and TNF superfamily member 4 (TNFSF4) displayed a strikingly high degree of statistical significance (*P* < 0.001) (Fig. 6c).

### Validation of the risk prognosis model

**Fig. 7:**
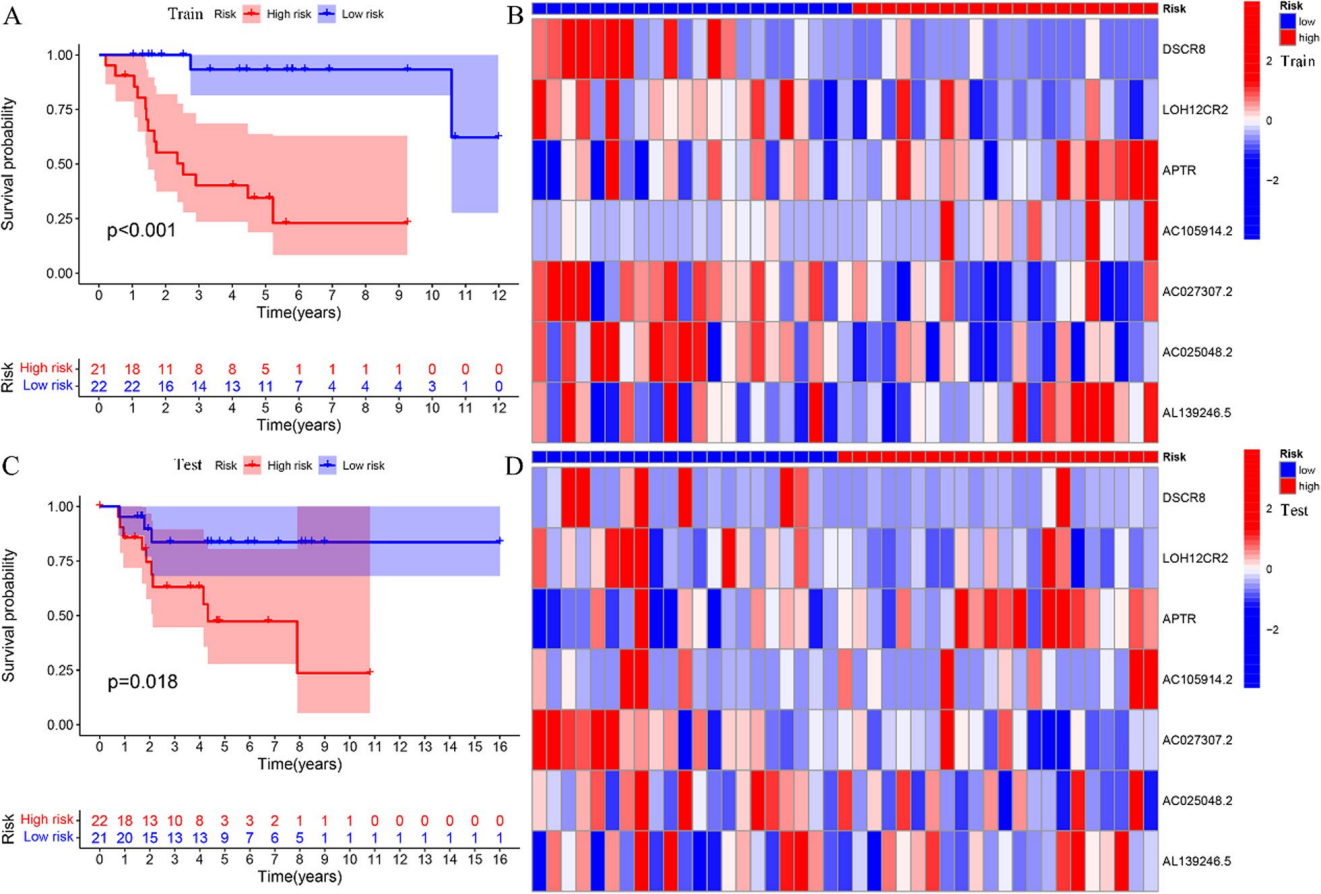
Validation of the risk prognosis model. **a** Survival analysis of train group. **b** Risk heatmap of train group. **c** Survival analysis of test group. **d** Risk heatmap of test group

Survival analyses were performed on both the train and test groups, revealing statistically significant differences in OS patient survival between high-risk and low-risk groups (Fig. 7a, c). The risk assessment heatmaps, generated for both the train and test groups, displayed variations in the expression levels of the seven FRLncs that were incorporated into the predictive model. Specifically, the expression levels of APTR, AC105914.2 and AL139246.5 exhibited a progressive increase from the low-risk to the high-risk group, thereby categorizing them as high-risk FRLncs. Conversely, DSCR8, LOH12CR2, AC027307.2 and AC025048.2 displayed a consistent decline in expression levels, signifying their classification as low-risk FRLncs (Fig. 7b, d).

## Discussion

We developed a risk prognostic model for assessing the OS-related FRLncs based on a comprehensive analysis of data from both TCGA and GEO databases. This model incorporates information from seven FRLncs classified into two categories: three high-risk FRLnc and four low-risk FRLncs. The risk prognosis model constructed in this study can well predict the survival of patients with OS, and has a certain impact on the immune microenvironment of patients with OS. Compared with the low-risk group, the immune cells and immune function of patients with OS in the high-risk group showed a downward trend. Immune checkpoint-related genes also differed between the two group.

Previous study has shown that LncRNA APTR participates in the progression of OS by repression of miR-132-3p and upregulation of Yes-associated protein 1 (YAP1) [23]. The expression of APTR in OS tumor tissues and four OS cell lines (MG63, 143B, Saos-2 and HOS) was significantly up-regulated compared with that of in neighboring tissues and human osteoblast cell lines hFOB1.19, respectively. MiR-132-3p is the target of APTR, and its expression is inhibited by APTR. Both knockdown of APTR and overexpression of miR-132-3p can significantly inhibit the proliferation, invasion and migration of human OS cells, and induce cell apoptosis. In addition, YAP1 was identified as a target for the inhibition of miR-132-3p [23]. LncRNA DSCR8 promotes the proliferation of liver cancer cells and inhibits apoptosis via the miR-22-3p/ARPC5 axis [24]. LncRNA DSCR8 mediates miR-137/Cdc42 to regulate gastric cancer cell proliferation, invasion and cell cycle as a competitive endogenous RNA [25]. The study found that smoking promoted the development of lung adenocarcinoma and lung squamous cell carcinoma by affecting gender-specific lncRNA changes. In women with lung squamous cell carcinoma, changes in LOH12CR2 were positively associated with smoking index [26]. AC027307.2 can not only be used as an enhancer-associated lncRNA to become a specific prognostic marker for breast cancer, but also as an immune-associated lncRNA to predict the survival rate of patients with colorectal adenocarcinoma [27, 28]. Notably, of the seven FRLncs that can guide OS prognosis and immunity, APTR, DSCR8, LOH12CR2 and AC027307.2 were all associated with tumor progression and prognosis. Among them, APTR has been found to be involved in the development of OS in previous studies. These findings underscore the robustness of our results. In the existing literature, AC105914.2, AL139246.5 and AC025048.2 remain to be elucidated and documented.

The immune system was one of the major components in the tumor microenvironment and was often suppressed in hypoxia [29]. The study found that the pre-treatment neutrophil-to-lymphocyte ratio was of diagnostic and prognostic value for OS [30]. Clinical data suggest that NK cells may play an important role in the prevention and therapeutic response of OS. In patients with OS, the number of circulating NK cells in peripheral blood was lower than in normal controls, suggesting that NK cells play a preventive role in the development of OS tumors [31]. pDCs exhibit remarkable proficiency in the rapid and robust production of type I interferon (IFN-I/α) [32]. Nevertheless, in the context of cancer, pDCs demonstrate a diminished responsiveness to Toll-like receptor 7 and 9 (TLR7/9) activation, resulting in a marked reduction or outright loss of IFN-α production. This diminished IFN-α contributes to the establishment of an immunosuppressive tumor microenvironment [33]. Notably, pDCs play a pivotal role in the regulation of both innate and adaptive immune systems, rendering them essential actors in the realm of cancer immunity [33]. Investigations focusing on the interplay between immune cell populations and the OS microenvironment have elucidated that cancer progression is most expedited when anti-tumor immune cells, including DCs, helper T cells, cytotoxic cells and IFN-γ, exhibit a decline in abundance, while regulatory T cells (Treg) undergo an increase [34]. Several studies have posited that the secretion of cytokines and the proliferative capacity of T follicular helper (Tfh) cells are markedly impaired in OS patients. This diminishment in functionality may underlie the body's compromised ability to resist OS development [35]. Importantly, miR-138 serves as an inhibitor of the PI3K/Akt/mTOR pathway by specifically targeting and negatively regulating pyruvate dehydrogenase kinase 1 (PDK1), thereby mitigating Tfh dysfunction in OS [36]. The TIL tasked with combating tumor cells within the OS microenvironment experience depletion, thus hastening tumor recurrence [37]. Notably, the incorporation of adjuvant chemotherapy in conjunction with TIL therapy has demonstrated the capacity to extend survival in OS patients with a suboptimal response to neoadjuvant chemotherapy [37]. Within the cancer microenvironment, innate immunity is systematically suppressed in the context of immune tolerance. A pivotal mechanism underlying immune tolerance is the immune checkpoint mechanism, which operates to restrain T cell activity in order to prevent undue immune responses [38].

The high-risk group exhibited significant down-regulation of immune functions, including APC-co-stimulation, immune checkpoint regulation, cytolytic activity and T cell co-inhibition. Study revealed that the administration of the anti-epidermal growth factor receptor (EGFR) antibody, cetuximab, resulted in an augmentation of the cytolytic activity exhibited by NK cells in the context of OS [39]. Immune checkpoint inhibitors (ICIs) have been recognized for their capacity to reshape the course of various malignancies by eliciting a disruption in immune homeostasis, thereby empowering the host's immune system to combat the tumor [40]. In a comprehensive analysis focusing on hypoxic prognostic indicators associated with OS metastasis and immune cell infiltration, a pronounced down-regulation of immune checkpoint mechanisms was identified among high-risk populations [29]. Regrettably, the precise correlation between APC-co-stimulation and T cell co-inhibition with respect to OS remains an area of investigation that warrants further elucidation in the scientific literature.

CD200R1 is not only differentially expressed in non-small cell lung cancer and has a prognostic effect, but also predicts survival in patients with head and neck squamous cell carcinoma [41, 42]. Previous study has found that HAVCR2 can be used as immune signature to accurately predict the prognosis of patients with OS, high expression of HAVCR2 is associated with improved prognosis [43, 44]. A comprehensive investigation has revealed significant alterations in Galectin-9, encoded by the LGALS9, across various cancer types [45]. Remarkably, LGALS9 not only exhibits associations with mRNA expression levels in cervical cancer cells but also emerges as a potential prognostic biomarker in pancreatic cancer [46]. The CD70-CD27 interaction is important for the regulation of adaptive immunity. Phospholipase C epsilon 1(PLCE1) is a marker of poor prognosis and may promote immune escape of OS through the CD70-CD27 signaling pathway [47]. LAIR1 overexpression inhibits the epithelial–mesenchymal transformation of OS through glucose transporter (Glut) 1-related energy metabolism [48]. The construction and validation of an oxidative stress-related prognostic risk model for OS suggests that LAG3 is a potential immunotherapeutic target for patients with OS [49]. In all melanoma patients and in the stage III and IIIc-IV patient cohorts, low expression of TNFSF4 was associated with a poorer prognosis. In the subgroup of patients with low lymphocytic infiltration, low expression of TNFSF4 was also associated with a poorer prognosis [50]. It is suggested that TNFSF4 is associated with tumor prognosis.

Nevertheless, it is imperative to acknowledge the presence of several limitations within the scope of this investigation. Firstly, the study's sample size remained relatively small, and the sampling methodology employed did not successfully mitigate the potential confounding influence of gender and underlying medical conditions. Secondly, the FRLncs identified in this study, which bear the potential to prognostic outcomes in the context of OS and contribute insights into the immune microenvironment, have not yet undergone experimentally validation. However, it is noteworthy that this validation process constitutes a focal point for our forthcoming research endeavors.

## Conclusion

In this study, a prognostic risk model for OS was formulated by integrating data from the TCGA and GEO databases. Concurrently, immune analysis was conducted, yielding seven FRLncs identified as potential prognostic markers for OS and influencers of the immune microenvironment. Differential profiles of immune cells and functional characteristics were delineated within the context of the risk prognostic model. Furthermore, we identified seven immune checkpoint-associated genes with notable implications for OS prognosis. The discovery of these FRLncs, their pivotal roles in OS prognosis, the modulation of the immune microenvironment, as well as the identification of immune checkpoint-related genes, collectively furnish a solid theoretical foundation for advancing research in OS survival and facilitating informed clinical decision making.

### Supplementary Information

**Additional file 1**. **Table 1**: ferroptosis-related genes. **Table 2**: differentially expressed genes of GSE19276 dataset. **Table 3**: differentially expressed genes of GSE16088 and GSE33382 datasets.

## Data Availability

The data supporting the results of the study are available from the TCGA database (https://portal.gdc.cancer.gov/) and GEO database (https://www.ncbi.nlm.nih.gov/geo/).

## References

[1] Zhu Y, Zhou J, Ji Y, Yu B (2014). Elevated expression of AKT2 correlates with disease severity and poor prognosis in human osteosarcoma. Mol Med Rep.

[2] Jaffe N (2009). Adjuvant chemotherapy in osteosarcoma: an odyssey of rejection and vindication. Cancer Treat Res.

[3] Bu X, Liu J, Ding R, Li Z (2021). Prognostic value of a pyroptosis-related long noncoding RNA signature associated with osteosarcoma microenvironment. J Oncol.

[4] Kong C, Hansen MF (2009). Biomarkers in osteosarcoma. Expert Opin Med Diagn.

[5] Chen Y, Tang G, Qian H, Chen J, Cheng B, Zhou C (2021). LncRNA LOC100129620 promotes osteosarcoma progression through regulating CDK6 expression, tumor angiogenesis, and macrophage polarization. Aging (Albany NY).

[6] Huang S, Zhu X, Ke Y, Xiao D, Liang C, Chen J (2020). LncRNA FTX inhibition restrains osteosarcoma proliferation and migration via modulating miR-320a/TXNRD1. Cancer Biol Ther.

[7] Su Y, Zhao B, Zhou L, Zhang Z, Shen Y, Lv H (2020). Ferroptosis, a novel pharmacological mechanism of anti-cancer drugs. Cancer Lett.

[8] Qiu Y, Cao Y, Cao W, Jia Y, Lu N (2020). The application of ferroptosis in diseases. Pharmacol Res.

[9] Ma S, Henson ES, Chen Y, Gibson SB (2016). Ferroptosis is induced following siramesine and lapatinib treatment of breast cancer cells. Cell Death Dis.

[10] Eling N, Reuter L, Hazin J, Hamacher-Brady A, Brady NR (2015). Identification of artesunate as a specific activator of ferroptosis in pancreatic cancer cells. Oncoscience.

[11] Liang C, Zhang X, Yang M, Dong X (2019). Recent progress in ferroptosis inducers for cancer therapy. Adv Mater.

[12] Shi Y, Gong M, Deng Z, Liu H, Chang Y, Yang Z (2021). Tirapazamine suppress osteosarcoma cells in part through SLC7A11 mediated ferroptosis. Biochem Biophys Res Commun.

[13] Lin H, Chen X, Zhang C, Yang T, Deng Z, Song Y (2021). EF24 induces ferroptosis in osteosarcoma cells through HMOX1. Biomed Pharmacother.

[14] Lei T, Qian H, Lei P, Hu Y (2021). Ferroptosis-related gene signature associates with immunity and predicts prognosis accurately in patients with osteosarcoma. Cancer Sci.

[15] Cai W, Li H, Zhang Y, Han G (2020). Identification of key biomarkers and immune infiltration in the synovial tissue of osteoarthritis by bioinformatics analysis. PeerJ.

[16] Lu L, Liu LP, Zhao QQ, Gui R, Zhao QY (2021). Identification of a ferroptosis-related LncRNA signature as a novel prognosis model for lung adenocarcinoma. Front Oncol.

[17] Endo-Munoz L, Cumming A, Sommerville S, Dickinson I, Saunders NA (2010). Osteosarcoma is characterised by reduced expression of markers of osteoclastogenesis and antigen presentation compared with normal bone. Br J Cancer.

[18] Fan Q, Liu B (2016). Identification of a RNA-Seq based 8-long non-coding RNA signature predicting survival in esophageal cancer. Med Sci Monit.

[19] Shannon P, Markiel A, Ozier O, Baliga NS, Wang JT, Ramage D (2003). Cytoscape: a software environment for integrated models of biomolecular interaction networks. Genome Res.

[20] Zhang Y, He R, Lei X, Mao L, Jiang P, Ni C (2021). A novel pyroptosis-related signature for predicting prognosis and indicating immune microenvironment features in osteosarcoma. Front Genet.

[21] Li J, Tang X, Du Y, Dong J, Zhao Z, Hu H (2021). Establishment of an autophagy-related clinical prognosis model for predicting the overall survival of osteosarcoma. Biomed Res Int.

[22] Jiang F, Miao XL, Zhang XT, Yan F, Mao Y, Wu CY (2021). A hypoxia gene-based signature to predict the survival and affect the tumor immune microenvironment of osteosarcoma in children. J Immunol Res.

[23] Guan H, Shang G, Cui Y, Liu J, Sun X, Cao W (2018). Long noncoding RNA APTR contributes to osteosarcoma progression through repression of miR-132-3p and upregulation of yes-associated protein 1. J Cell Physiol.

[24] Huang J-N, Zhang H-M, Cai J-D, Wang W-L, Wang P (2023). Long noncoding RNA DSCR8 promotes the proliferation of liver cancer cells and inhibits apoptosis via the miR-22-3p/ARPC5 Axis. J Cancer.

[25] Chen Z, Xu C, Pan X, Cheng G, Liu M, Li J (2021). lncRNA DSCR8 mediates miR-137/Cdc42 to regulate gastric cancer cell proliferation, invasion, and cell cycle as a competitive endogenous RNA. Mol Therapy Oncol.

[26] Cheng Z, Liu B, Liu Y, Zou J, Zou M (2022). Smoking is associated with lung adenocarcinoma and lung squamous cell carcinoma progression through inducing distinguishing lncRNA alterations in different genders. Anticancer Agents Med Chem.

[27] Zhao H, Zhang S, Yin X, Zhang C, Wang L, Liu K (2022). Identifying enhancer-driven subtype-specific prognostic markers in breast cancer based on multi-omics data. Front Immunol.

[28] Li Z, Wang D, Yin H (2020). A seven immune-related lncRNA signature predicts the survival of patients with colon adenocarcinoma. Am J Transl Res.

[29] Fu Y, Bao Q, Liu Z, He G, Wen J, Liu Q (2021). Development and validation of a hypoxia-associated prognostic signature related to osteosarcoma metastasis and immune infiltration. Front Cell Dev Biol.

[30] Yapar A, Tokgoz MA, Yapar D, Atalay IB, Ulucakoy C, Gungor BS (2021). Diagnostic and prognostic role of neutrophil/lymphocyte ratio, platelet/lymphocyte ratio, and lymphocyte/monocyte ratio in patients with osteosarcoma. Jt Dis Relat Surg.

[31] Tarek N, Lee DA (2014). Natural killer cells for osteosarcoma. Adv Exp Med Biol.

[32] Reizis B (2019). Plasmacytoid dendritic cells: development, regulation, and function. Immunity.

[33] Mitchell D, Chintala S, Dey M (2018). Plasmacytoid dendritic cell in immunity and cancer. J Neuroimmunol.

[34] Le T, Su S, Kirshtein A, Shahriyari L (2021). Data-driven mathematical model of osteosarcoma. Cancers (Basel)..

[35] Gong L, Bao Q, Hu C, Wang J, Zhou Q, Wei L (2018). Exosomal miR-675 from metastatic osteosarcoma promotes cell migration and invasion by targeting CALN1. Biochem Biophys Res Commun.

[36] Jiang B, Kang X, Zhao G, Lu J, Wang Z (2021). miR-138 reduces the dysfunction of T follicular helper cells in osteosarcoma via the PI3K/Akt/mTOR pathway by targeting PDK1. Comput Math Methods Med.

[37] Shi J, Li M, Yang R (2020). Tumor-infiltrating lymphocytes as a feasible adjuvant immunotherapy for osteosarcoma with a poor response to neoadjuvant chemotherapy. Immunotherapy.

[38] Yoshida K, Okamoto M, Sasaki J, Kuroda C, Ishida H, Ueda K (2020). Anti-PD-1 antibody decreases tumour-infiltrating regulatory T cells. BMC Cancer.

[39] Pahl JH, Ruslan SE, Buddingh EP, Santos SJ, Szuhai K, Serra M (2012). Anti-EGFR antibody cetuximab enhances the cytolytic activity of natural killer cells toward osteosarcoma. Clin Cancer Res.

[40] Guven DC, Kilickap S, Yildirim HC, Ceylan F, Bas O, Dizdar O (2021). Chemoimmunotherapy for the salvage treatment of Ewing sarcoma: a case report. J Oncol Pharm Pract.

[41] Yoshimura K, Suzuki Y, Inoue Y, Tsuchiya K, Karayama M, Iwashita Y (2020). CD200 and CD200R1 are differentially expressed and have differential prognostic roles in non-small cell lung cancer. OncoImmunology..

[42] Chang H, Lee Y-G, Ko YH, Cho JH, Choi J-K, Park KU (2020). Prognostic value of CD200R1 mRNA expression in head and neck squamous cell carcinoma. Cancers.

[43] Yang J, Zhang A, Luo H, Ma C (2022). Construction and validation of a novel gene signature for predicting the prognosis of osteosarcoma. Sci Rep.

[44] Song YJ, Xu Y, Deng C, Zhu X, Fu J, Chen H (2021). Gene Expression classifier reveals prognostic osteosarcoma microenvironment molecular subtypes. Front Immunol.

[45] Armenta-Castro E, Reyes-Vallejo T, Maximo-Sanchez D, Herrera-Camacho I, Lopez-Lopez G, Reyes-Carmona S (2020). Histone H3K9 and H3K14 acetylation at the promoter of the LGALS9 gene is associated with mRNA levels in cervical cancer cells. FEBS Open Bio.

[46] Fan Y, Li T, Xu L, Kuang T (2020). Comprehensive analysis of immunoinhibitors identifies LGALS9 and TGFBR1 as potential prognostic biomarkers for pancreatic cancer. Comput Math Methods Med.

[47] Huang L, Liao C, Wu H, Huang P (2022). PLCE1 is a poor prognostic marker and may promote immune escape from osteosarcoma by the CD70-CD27 signaling pathway. Bosn J Basic Med Sci.

[48] Zhang J, Zhang Y, Cheng S, Mu Y, Liu Y, Yi X (2020). LAIR-1 overexpression inhibits epithelial-mesenchymal transition in osteosarcoma via GLUT1-related energy metabolism. World J Surg Oncol.

[49] Wang H, Li J, Li X (2023). Construction and validation of an oxidative-stress-related risk model for predicting the prognosis of osteosarcoma. Aging (Albany NY).

[50] Roszik J, Markovits E, Dobosz P, Layani A, Slabodnik-Kaner K, Baruch EN (2019). TNFSF4 (OX40L) expression and survival in locally advanced and metastatic melanoma. Cancer Immunol Immunother.

